# Proposal of *Rotundianchoratus dactyliriformis* gen. nov. sp. nov. and two new species of *Urocleidoides* (Monopisthocotyla, Dactylogyridae) parasitizing the gills of *Hemiodus unimaculatus* (Characiformes, Hemiodontidae)

**DOI:** 10.1007/s11230-026-10304-9

**Published:** 2026-10-28

**Authors:** Álvaro José Bittencourt de Freitas, Marcia Cristina Nascimento Justo, Diego Carvalho Viana, Simone Chinicz Cohen

**Affiliations:** 1 Laboratório de Helmintos Parasitos de Peixes – LHPP, Instituto Oswaldo Cruz, Fiocruz. Avenida Brasil, 4365, Manguinhos, Rio de Janeiro, RJ 21040-360 Brazil; 2 Núcleo de Estudos Morfofisiológicos Avançados – NEMO, Universidade Estadual da Região Tocantina do Maranhão, Campus Imperatriz, Rua Godofredo Viana, 1300, Imperatriz, MA 30065901-480 Brazil

## Abstract

*Rotundianchoratus* **gen. nov.** is proposed to accommodate a new species of dactylogyrid monopisthocolylan parasitizing the gills of *Hemiodus unimaculatus* (Characiformes: Hemiodontidae) from the Tocantins–Araguaia River basin, Brazil. *Rotundianchoratus* **gen. nov.** is mainly characterized by the presence of a well-developed protuberance at the middle shaft of the ventral anchor, a feature not previously reported for other genera within Dactylogyridae. The new genus also presents a tubular male copulatory organ (MCO) with one to two counterclockwise rings, an accessory piece articulated by a copulatory ligament, and a sclerotized vagina. This genus can be distinguished from other genera by the combination of the morphology of the copulatory complex and the unique structure of the anchors, particularly the conspicuous mid-shaft protuberance on the ventral anchor. Additionally, two new species of *Urocleidoides* Mizelle & Price, 1964 are described from the same host species. Both species are characterized by the presence of a copulatory ligament, a sclerotized vagina, and a distinct hook configuration, with pairs 6 and 7 larger than the remaining pairs. The new species differ from their congeners by features of the copulatory complex and structures of the haptor. This study represents the first record of monopisthocolylans infecting *H. unimaculatus* and expands the knowledge of dactylogyrid diversity in Neotropical freshwater systems. The findings also reinforce the morphological variability within *Urocleidoides* and highlight the importance of detailed morphological analyses for the recognition of taxonomic diversity within Dactylogyridae.

## Introduction

The continuous discovery of new fish species highlights the incomplete knowledge of global ichthyofauna. Over 200 species were recognized annually until 2006 and continued growth, reaching 407 new taxa in 2024 (Eschmeyer et al., 2018; Froese & Pauly, 2026; Fricke et al., 2026). It is recognized that there are about 6,200 fish species in the Neotropical Region, which hosts 20–25% of global freshwater fish diversity, with Characiformes—a highly diverse order compressing over 2,000 taxa, playing key ecological and economic roles in South America and Africa (Reis et al., 2016; Albert et al., 2020; Froese & Pauly, 2026).

The Tocantins–Araguaia River basin harbors a rich and diverse ichthyofauna, with more than 700 species recorded, representing a significant portion of Brazilian freshwater biodiversity (Chicrala et al., 2013). Despite its high diversity, this basin has been increasingly impacted by anthropogenic activities, including dam construction, agriculture, and mining (Chamon et al., 2022). Within this system, the family Hemiodontidae comprises medium-sized, fusiform fishes that are widely distributed in Neotropical rivers, including *Hemiodus unimaculatus* (Bloch), an ecologically important species in these environments. (Langeani, 2004; Froese & Pauly, 2026).

The genus *Urocleidoides* Mizelle & Price, 1964 currently comprises approximately 60 valid species parasitizing Neotropical fishes. The taxonomy of this genus has undergone several revisions based on morphological and, more recently, molecular data, which have expanded the diagnostic boundaries and revealed considerable morphological diversity within the group (Kritsky et al., 1986; Santos-Neto & Domingues, 2023; Osaki-Pereira et al., 2025).

Despite recent advances, records of monopisthocolylans infecting *H. unimaculatus* remain scarce, with previous studies reporting mainly Nematoda, Digenea, and Myxozoans. During studies on monopisthocolylan fauna parasitizing *H. unimaculatus* from the Tocantins River basin, Brazil, a new dactylogyrid genus and two new species of *Urocleidoides* are proposed herein, thereby expanding current knowledge of parasite diversity and host–parasite associations in Neotropical freshwater ecosystems.

## Materials and methods

A total of 134 specimens of *H. unimaculatus* were collected along the riverside within the urban zone of Imperatriz municipality, Maranhão State, Brazil (5°31′47″S, 47°29′30″W). The specimens were captured with the assistance of local fishermen with “set-net”. The specimens were transported to the “Núcleo de Estudos Morfológicos Avançados (NEMO)” at the “Universidade Estadual da Região Tocantina do Maranhão” (UEMASUL), where taxonomic identification was performed. The collection license was obtained from the System for Authorization and Information on Biodiversity (SISBIO) under protocol number 69275-1. The gills of fish were removed and placed in vials containing hot water (~65°C) and vigorously shaken to detach the parasites from the gill filaments. Subsequently, absolute ethanol was added to reach a concentration of 70%. Those vials containing the gills were then sent to the “Laboratório de Helmintos Parasitos de Peixes, Instituto Oswaldo Cruz, FIOCRUZ”, Rio de Janeiro State, Brazil. The gills and sediments were analyzed in Petri dishes under a stereoscopic microscope, and the specimens were mounted in Hoyer’s medium to study the sclerotized structures (Boeger & Vianna, 2006).

Parasitological indices, as prevalence, mean abundance and mean intensity, were calculated according to Bush et al. (1997). Range of intensity is presented for each species. Measurements are presented in micrometers, range values are followed by the mean and number of structures measured in parentheses. Photomicrographs and drawings were taken using, respectively, a Zeiss^®^ Axioskop microscope micrographic system with a differential interference contrast (DIC) apparatus and an Olympus BX 41 microscope with phase contrast, equipped with a camera lucida. Holotype, paratypes, and vouchers were deposited in the “Coleção Helmintológica do Instituto Oswaldo Cruz - CHIOC” in Brazil. Morphometric studies were made with assistance of ImageJ software for irregular structures, distributed by the National Institutes of Health (NIH) (Wayne, 2010).

Authorship and nomenclatural acts for taxa were identified following the provisions of Article 50.1 and Recommendation 50A of the International Code of Zoological Nomenclature (ICZN), which deal specifically with the attribution of authorship.

For comparative study, types of *Urocleidoides luquei* Hasuike Scorsim, Arjona, Bellay, de Oliveira & Takemoto, 2024 numbers 40270 (holotype), 40270 b-j (paratype); *Urocleidoides tocantinensis* Freitas, Bezerra, Meneses, Justo, Viana & Cohen, 2021 numbers 39575 (holotype), 39576a-b, 39577, 39578 (paratypes) and *Urocleidoides boulengerellae* Freitas, Bezerra, Meneses, Justo, Viana & Cohen, 2021 numbers39560 (holotype), 39561, 39562a-c, 39563a-b, 39564a-b, 39565, 39566 (paratypes) deposited in CHIOC were examined.

Results

Class Monopisthocotyla Brabec, Salomaki, Kolısko, Scholz & Kuchta, 2023

Order Dactylogyridea Bychowsky, 1937

Dactylogyridae Bychowsky, 1933

*Rotundianchoratus* **gen. nov**. Freitas, Justo & Cohen

Type species: *Rotundianchoratus dactyliriformis* **gen. et sp. nov.** Freitas, Justo & Cohen

Etymology: The name of the new genus is derived from Latin (*rotundus* = round; *anchoratus* = anchor) and it is given by the presence of a protuberance in the shaft of the ventral anchor. The genus name is masculine and should be treated as such.

**Diagnosis**. Body divided into cephalic region, trunk, peduncle and haptor. Tegument smooth. Cephalic lobes moderately developed with four pairs of bilateral head organs. Cephalic glands not observed. Two pairs of eyespots present, being the anterior small than the posterior one. Mouth subterminal, midventral; Pharynx muscular. Esophagus long. Intestinal caeca confluent posterior to testis, lacking diverticula. Vas deferens straight in the middle of the body. Vagina present, sclerotized, dextral, vaginal canal sclerotized. Germarium ovate or subovate, almost size of testis. Testis dorsal to germarium. Seminal vesicle ovate and prostatic reservoir thin and elongated. Copulatory complex comprises (MCO), accessory piece and copulatory ligament connecting them. Accessory piece sickle-shaped, robust. Copulatory ligament connecting MCO base to accessory piece in the middle portion. MCO a counterclockwise tube with two rings, base robust, finger-shaped. Peduncle short. Haptor subglobose, ventral and dorsal bars rod-shaped. Anchors dissimilar in shape; Ventral anchor with expansion in the middle of shaft point and long tip. Dorsal anchor with poorly developed protuberance in the middle of shaft point and long tip. Fourteen hooks with Ancyrocephaline distribution, composed by two subunits, length of subunits differing in pairs 1 and 5, recurved shaft point, thumb subsquare. Parasite of Neotropical characiforms (Hemiodontidae).

**Remarks** The new genus possesses a copulatory ligament, characteristic observed in some genera *e.g Boegeriella* Mendoza-Palmero & Hsiao, 2020, and *Philocorydoras* Suriano, 1986. However, the new genus can be distinguished from all other dactylogyrid genera by the presence of a well-developed protuberance in the middle of the shaft of the ventral anchor, and a poorly developed protuberance in the dorsal anchor. The protuberance in the middle of the anchors was only observed in the diplectanid *Rhabdosynochus hudsoni* Kritsky, Boeger & Robaldo, 2001, marine parasite of fish.

**Fig. 1:**
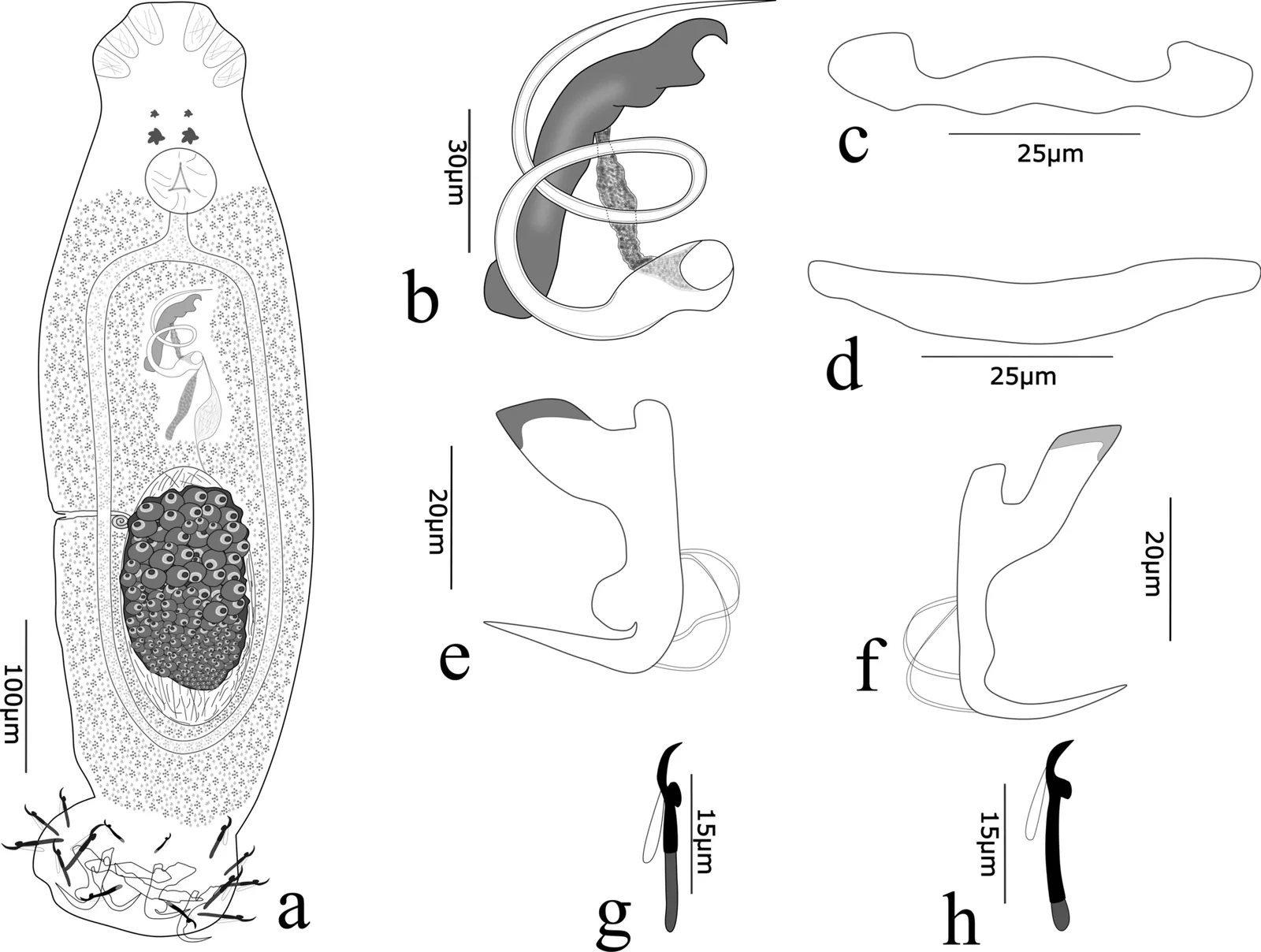
*Rotundianchoratus dactyliriformis* **gen et sp. nov.** parasite of *Hemiodus unimaculatus*. a: Total, ventral view (composite); b: Copulatory complex; c: Ventral bar; d: Dorsal bar; e: Ventral anchor. f: Dorsal anchor; g: Hooks pair 1,5; h: Hooks pairs 2–4, 6, 7

*Rotundianchoratus dactyliriformis* **gen. et sp. nov.** Freitas, Justo & Cohen (Fig. 1)

**Type host:** *Hemiodus unimaculatus* (Bloch)

**Site of infection:** Gills

**Type locality:** Tocantins River, near the urban perimeter of Imperatriz, in the community of Embiral, state of Maranhão, Brazil (5°27′50″S, 47°33′48″W)

**Infestation parameters:** Prevalence: 8.95% (12/134 infested); Mean abundance: 0.23; Mean intensity: 2; Range of intensity: 1–5

Etymology: The species name is from Greek (*Dactylo* = Finger) and Latin (*Formi*s = shape) and it refers to the base of the copulatory complex.

Deposited Material: CHIOC Holotype: CHIOC 41147; paratype: CHIOC 41148 (a-c); CHIOC 41149

Description (Based on 13 specimens mounted on Hoyer’s medium and 5 stained in Gomori´s trichrome): Body elongated, 468 (375–580; n = 11) long by 124 (75–162; n = 11) wide. Tegument smooth. Three pairs of poorly developed cephalic lobes, four pairs of head organs,cephalic glands not observed. two pairs of eyespots anterior smaller than posterior. Pharynx spherical to sub-spherical, 26 (25–30; n = 5). Peduncle short. Haptor sub-hexagonal, presenting dorsal and ventral anchors and bars, 100 (80–125; n = 9) long. Ventral anchor with rounded deep root, rectangular superficial root with a cap, straight shaft, bearing a robust protuberance at midlength and long tip, 31 (25–36; n = 11) long and 21 (18–21; n = 11) base. Dorsal anchor with short and straight deep root and sub-rectangular superficial root, straight shaft with a poorly developed protuberance and long tip, 33 (23–35; n = 11) long and 16 (11–18; n = 11) wide; Ventral bar rod-shaped with enlarged terminations, directed upwards 45 (35–51; n = 11) and dorsal bar irregular rod-shaped 49 (38–55; n = 11). Hooks similar in shape and dissimilar in size, recurved shaft point, thumb subsquare, FH loop ½ of shaft length. Shank of hook composed by two subunits, length of subunits differing in pairs 1 and 5 about ½ of total length, pairs 2–4 and pairs 6–7 1/5 of total length. Hook pair 1: 16 (15–17; n = 11); pairs 2–4: 24 (22–25; n = 8); pair 5: 21 (20–23; n = 7); pair 6: 27 (26–30; n = 9) and pair 7: 28 (26–29; n = 9). MCO with 1 to 2 counterclockwise rings, single base, 28 (26–30; n = 9). Accessory piece 38 (28–43; n = 11) articulated to the MCO through a robust copulatory ligament. Testis dorsal to germarium. Seminal receptacle in the shape of an elongated pouch. Vagina strongly sclerotized, vaginal opening dextral, in the middle region of the body. Germarium sub ovate 65 (55–70; n = 3). Eggs not observed. Vitellaria densely present, absent in the reproductive organs.

**Remarks**: The new species proposed herein is distinguished from other dactylogyrid species by a set of unique morphological characters. Notably, the presence of a protuberance on the anchors represents a diagnostic feature that has not been reported for any previously described genus within the family. In addition, the new species is characterized by the morphology of the MCO, which forms one to two counterclockwise rings and by the presence of a copulatory ligament that articulates the accessory piece with the base of the MCO. This new species is closely related to *Boegeriella conica* (Mendoza-Palmero, Medoza-Franco, Acosta & Scholz, 2019) by the morphology of copulatory complex, especially regarding the presence of the copulatory ligament, but differs by the main diagnostic characteristic of the new species, *i.e.*, the presence of a protuberance in the anchors.

**Fig. 2:**
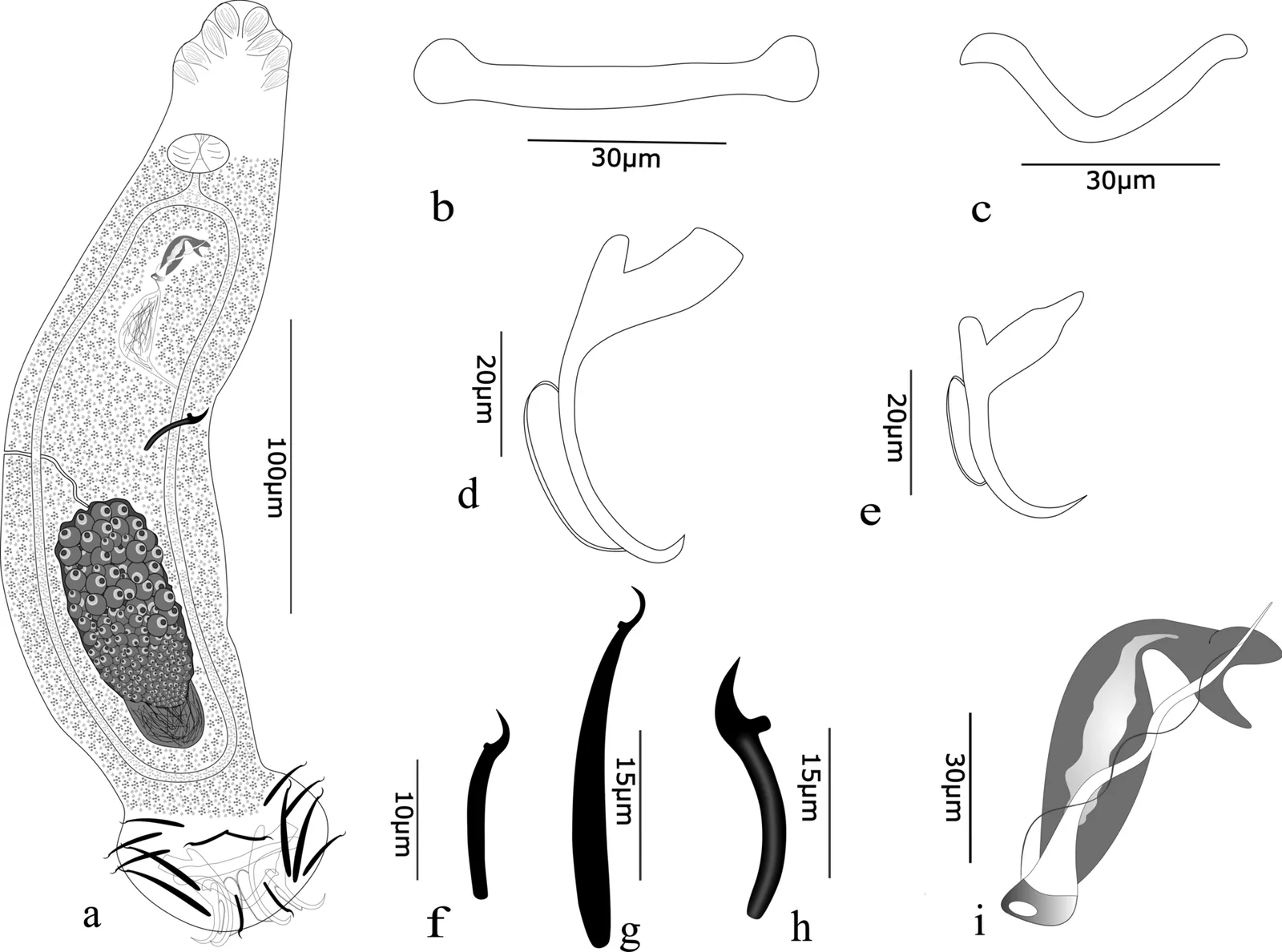
*Urocleidoides ichthyocercus* **sp. nov.** parasite of *Hemiodus unimaculatus*. a: Ventral view (composite). b: Ventral bar; c: Dorsal bar; d: Ventral anchor; e: Dorsal anchor; f: Hook pairs 1,5; g: Hook pairs 2,3,4,6,7; h: Vaginal sclerite; i: Copulatory complex

*Urocleidoides ichthyocercus* **sp. nov.** Freitas, Justo & Cohen (Fig. 2)

**Type host**: *Hemiodus unimaculatus* (Bloch)

**Type Locality**: Tocantins River, near the urban perimeter of Imperatriz; in the community of Embiral, state of Maranhão, Brazil (5°27′50″S, 47°33′48″W)

**Infestation parameters**: Prevalence: 6.71% (9/134 infested); Mean abundance: 0.14; Mean intensity: 2.1; Range of intensity: 1–9

**Etymology**: The specific name is from Greek (*ichthys* = fish; *cerco* = Tail) and it is given duo to the appearance of the copulatory complex, specific ally the accessory piece. Deposited material: Holotype: CHIOC 41150 (a); paratype: CHIOC 41150 (b); CHIOC 41151; CHIOC 41152

**Description** (Based on 19 specimens mounted on Hoyer’s medium): Body fusiform 442 (400–500; n = 6) long by 78 (60–90; n = 8) wide. Cephalic lobes poorly developed; four bilateral pairs of cephalic organs, eyespots absent. Pharynx muscular. Haptor sub globose. Ventral anchor robust with curved, rectangular-shaped superficial root and rounded deep root, slightly curved shaft, and short and curved tip, 51 (45–51; n = 10) long and 18 (15–20; n = 10) wide. Dorsal anchor with well-developed superficial root, narrowing towards the tip and rounded deep root, evenly curved shaft and straight tip, 30 (25–33; n = 10) long, base 12 (10–15; n = 10) wide. Ventral bar straight, with slightly enlarged ends, 48 (45–50; n = 10) long. Dorsal bar U-shaped, with straight ends, 43 (38–45; n = 10) long. Hooks similar in shape, with slightly developed thumb, recurved point; Pairs 1 and 5 reduced in size; Pair 1, 23 (22–25; n = 10) long, Pair 5, 19 (17–25; n = 6) long; Pairs 2–4, 33 (30–36; n = 9) long; pair 6, 43 (40–46; n = 10) and pair 7, 56 (50–62; n = 10) long. Copulatory complex composed of the male copulatory organ (MCO) connected to the accessory piece by a copulatory ligament. MCO comprises a sinuous tube, 30 (28–32; n = 8) long; accessory piece leaf-shaped with a slit part in distal position, robust, curved, 27 (23–30; n = 8). Canal vaginal slightly sclerotized, opening in the dextral position. Vaginal sclerite, robust, 35 (30–38; n = 7) long. Testis posterior to germarium, germarium subovate 70 (50–80; n = 3). Eggs, Mehlis’ glands and seminal vesicle not observed. Dense vitellaria distributed throughout body of the parasite, except in the region of the reproductive organs.

**Remarks**: The new species resembles *Urocleidoides luquei* Hasuike, Scorsim, Arjona, Bellay, Oliveira & Takemoto, 2024, a parasite of *Hemiodus orthonops* Eigenmann & Kennedy, by the morphology of the bars and anchors, in the absence of eyespots, vaginal pore in position dextral, the presence of copulatory ligament connecting MCO to accessory piece and in the size of the hooks (hooks size smaller in *U. luquei* and hook pairs 6 and 7 longer than the others in the new species). Both species can be distinguished by the morphology of the copulatory complex (MCO with ½ rings counterclockwise in *U. luquei* x MCO as a sinuous tube and accessory piece with two subunits in *Urocleidoides ichthyocercus* **sp. nov.**, the posterior one clamp-shaped in *U*. *luquei* x leaf-shaped with a slit in the middle in the new species)

**Fig. 3:**
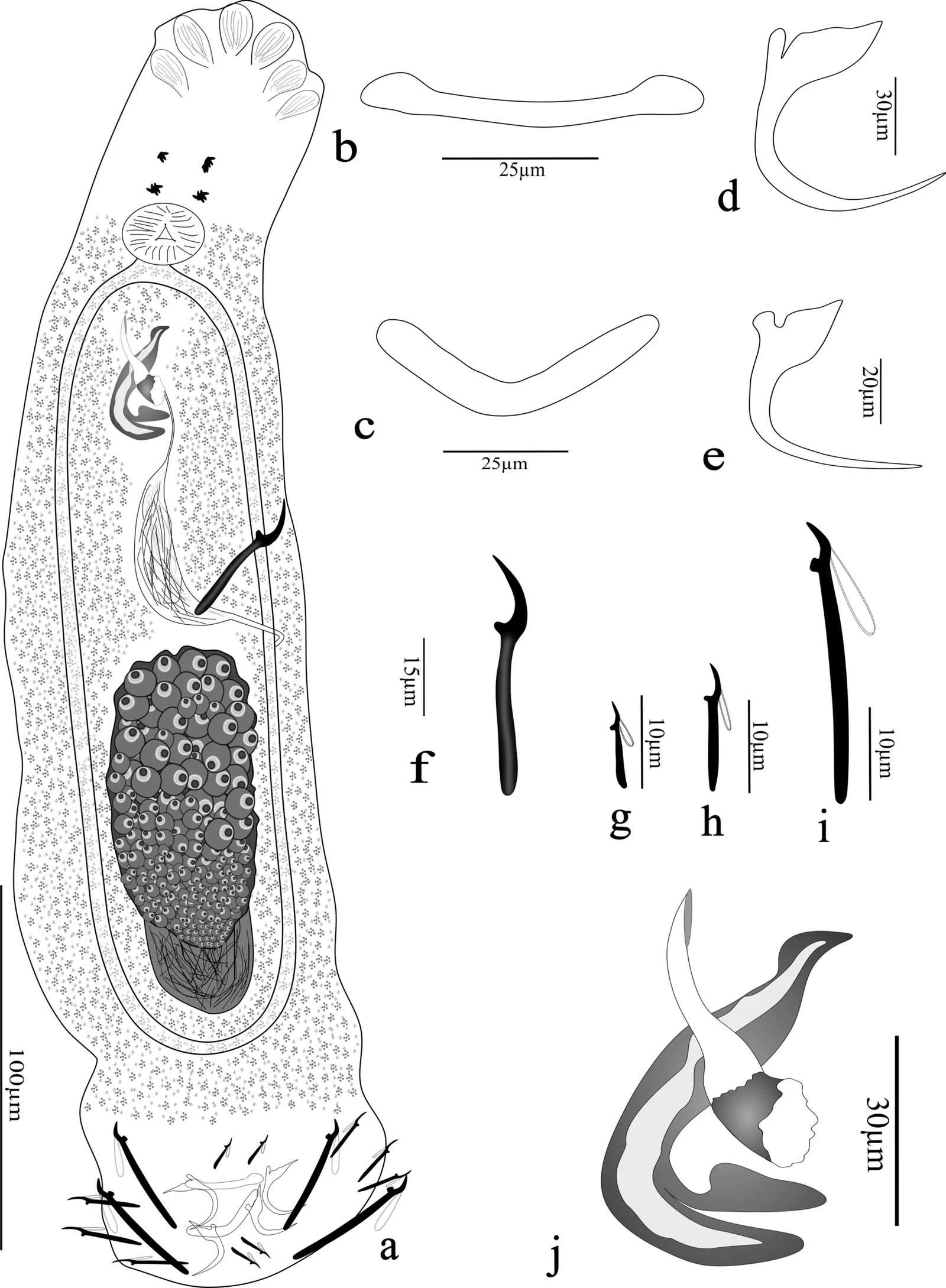
*Urocleidoides novaculatus* **sp. nov**. parasite of *Hemiodus unimaculatus*. A: Dorsal view (Composite). b: Ventral bar; c: Dorsal bar; d: Ventral anchor, e: Dorsal anchor; f: Vaginal sclerite; g: hooks pair 1 and 5; h: pair 6 and 7, i: Hooks: hooks 2, 3 and 4; j: Copulatory Complex

*Urocleidoides novaculatus* **sp. nov.** Freitas, Justo & Cohen (Fig. 3)

**Type host**: *Hemiodus unimaculatus* (Bloch)

**Type locality**: Tocantins River, near the urban perimeter of Imperatriz, in the community of Embiral, state of Maranhão, Brazil (5°27′50″S, 47°33′48″W)

**Infestation parameters**: Prevalence: 2.98% (4/134 infested); Mean abundance: 0.08; Mean Intensity: 3; Range of Intensity: 1–4

**Etymology**: The specific name is an adjective from Latin (*novacula* = razor) and it is due to the morphology of the accessory piece Deposited material: Holotype: CHIOC 41153 (a); paratype: CHIOC 41153 (b); CHIOC 41154 (a, b); CHIOC 41155 (a, b).

Description (Based on 9 specimens mounted on Hoyer’s medium): Body fusiform 325 (300–385; n = 7) long and 132 (90–150; n = 7) wide. Cephalic lobes poorly developed; two bilateral pairs of cephalic organs and one terminal pair. Two pairs of eyespots. Pharynx muscular, spherical. Haptor sub-hexagonal, 100 (60–112; n = 7). Hooks of dissimilar shape, with developed thumb, robust shank; Pairs 1 and 5 reduced in size, Pairs 6 and 7 slightly larger than pairs 2, 3 and 4. Pair 1, 14 (11–16; n = 6) long, Pair 5, 12 (9–14; n = 3) long; Pairs 2–4, 24 (22–27; n = 7) long; pair 6, 30 (28–35; n = 4) and pair 7, 31 (30–35; n = 4) long; FH Loop at about 1/3 of the total size. Ventral anchor with well developed superficial and deep roots, straight shaft, blade and recurved tip longer than the superficial root, 24 (20–31; n = 5) long, base 9 (8–10; n = 5). Dorsal anchor with well-developed superficial and deep root, straight shaft and slightly recurved tip, 18 (15–20 n = 5) long, base 7 (6–9; n = 5) wide. Dorsal bar V-shaped, 22 and 24 (n = 2) long. Ventral bar, with protruding curved ends, 29 (25–35; n = 5) in length. Copulatory complex composed of the male copulatory organ (MCO) and accessory piece. MCO short, robust, rod-shaped, with an irregular sclerotized base, 30 (26–32; n = 6). Accessory piece robust, recurved, leaf shaped bifurcated at distal portion, non-articulated to MCO, 40 (36–45; n = 6) long. Vaginal sclerite robust and with a well-formed protruding thumb, 33 (28–35; n = 6) long. Seminal vesicle present, with an elongated shape. Testis subovate, germarium ovate (30 and 35; n = 2), Mehlis’ glands, not observed and dense vitellaria are distributed throughout the body of the parasite, except in the region of the reproductive organs.

**Fig. 4:**
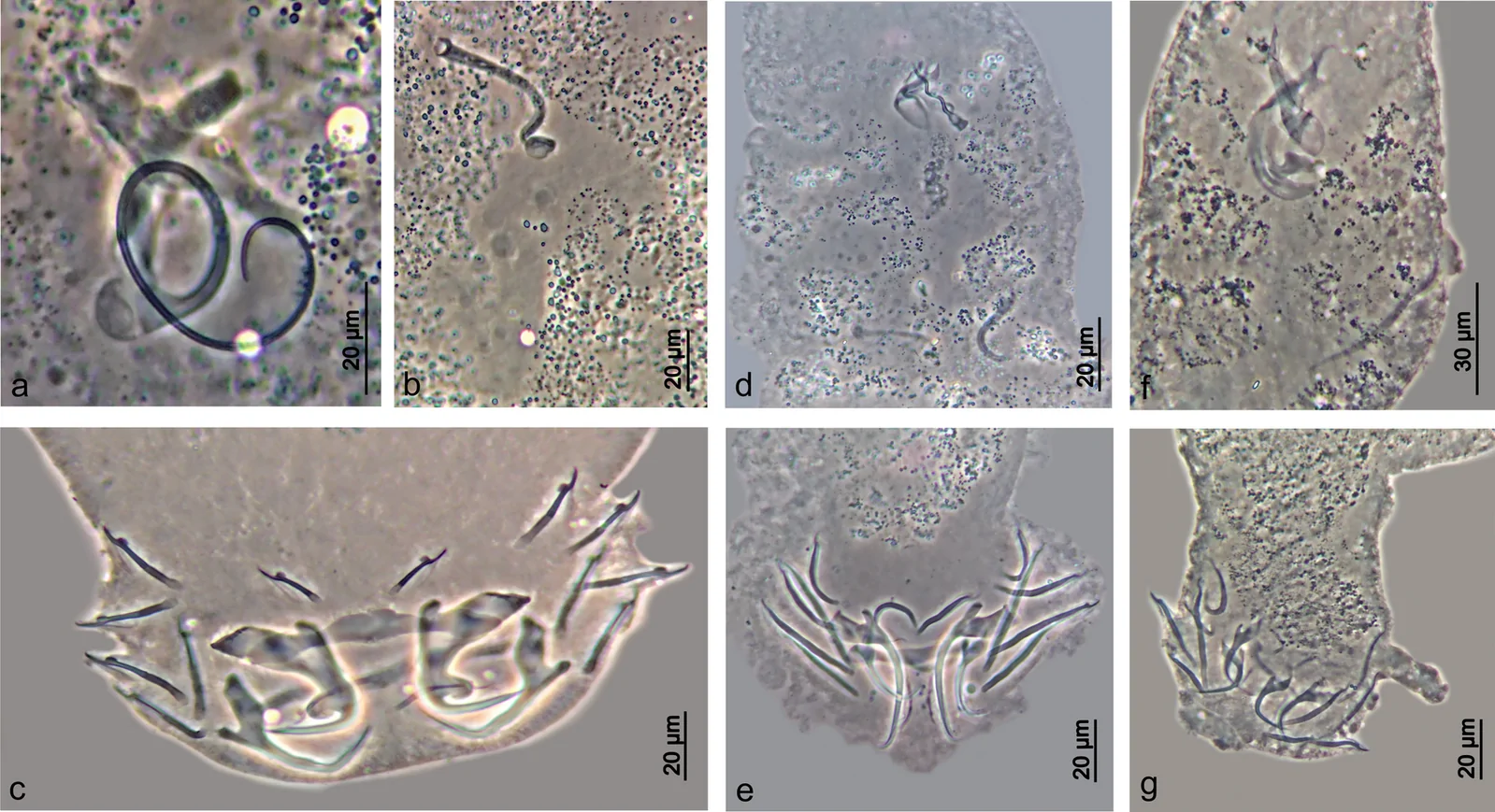
Photomicrographies of Monopisthocotyla parasites of *Hemiodus unimaculatus*. *Rotundianchoratus dactyliriformis* **gen. et sp. nov.** a: Copulatory complex; b: Vagina; c: Haptor. *Urocleidoides ichthyocercus* **sp. nov.** d: Copulatory complex, vagina and vaginal sclerite. e: Haptor. *Urocleidoides novaculatus* **sp. nov.** f: Copulatory complex, vagina and vaginal sclerite; g: Haptor

**Remarks**: This species resembles *Urocleidoides falxus* Zago, Yamada, Yamada, Franceschini, Bongiovani & da Silva, 2020, *Urocleidoides itabocaensis* Santos-Neto & Domingues, 2024, *Urocleidoides omphalocleithrum* Santos-Neto & Domingues, 2024, *Urocleidoides psectrogasteri* Bezerra, Freitas, Cohen & Justo, 2023, *Urocleidoides surianoae* Rossin & Timi, 2016, *Urocleidoides tocantinensis* Freitas, Bezerra, Meneses, Justo, Viana & Cohen, 2021 and *Urocleidoides ichthyocercus* **sp. nov.** by the absence of rings in the MCO, but differs from these species by the general morphology of the accessory piece, with fissure in the middle portion and by the morphology of the MCO, short, robust, resemblin a spine, characteristic not yet observed in this genus. The species *Urocleidoides ichthyocercus* **sp. nov.** and *Urocleidoides novaculatus* **sp. nov.** are closely related by the presence of hook pairs 6 and 7 larger than the others (Fig. 4).

## Discussion

The present study expands the knowledge of monopisthocotylan diversity associated with Hemiodontidae by providing the first record of a member of this group parasitizing *Hemiodus unimaculatus*. A new genus and species of Dactylogyridae is described, as well as two new species of *Urocleidoides*, reinforcing the high, yet still underestimated, diversity of monopisthocolylans in Neotropical freshwater systems.

To date, few species of Monopisthocotyla had been reported from members of the family Hemiodontidae as *Urocleidoides luquei* Hasuike, Scorsim, Arjona, Bellay, de Oliveira & Takemoto, 2024, parasite from *Hemiodus orthonops*; *Cleidodiscus microcirrus* Price & Schlueter, 1967 from *Hemiodus semitaeniatus* and *Monocleithrium lavergneae* Price & McMahon, 1966 from *Hemiodus semitaeniatus* (Price & McMahon, 1966, Price & Schlueter 1967, Hasuike et al., 2024). Additionally, in literature, reports on *H. unimaculatus* were only made for Nematoda and Digenea (*see* Cárdenas et al., 2022) and for Myxozoa (*see* Silva et al., 2020).

The morphological feature observed in the *Rotudianchoratus* **gen. nov.**, i.e. the presence of a well-developed protuberance located at the mid-shaft of the ventral anchor, was not previously reported for other genera within Dactylogyridae. Comparable structures have been observed in distantly related taxa, such as a marine monopisthocotylan, the diplectanid *R. hudsoni* Kritsky, Boeger & Robaldo, 2001. In addition to this unique feature, the copulatory complex exhibits similarities with those of *Diaphorocleidus* Jogunoori, Kritsky & Venkatanarasaiah, 2004 and *Boegeriella* Mendoza-Palmero & Hsiao, 2020, particularly in the general morphology of the accessory piece and the presence of a copulatory ligament. This combination of characters supports the establishment of a new genus and highlights the morphological diversity within Dactylogyridae.

*Urocleidoides* was originally proposed by Mizelle and Price (1964) which was characterized mainly by the presence of vaginal sclerite, hook 1 and 5 reduced and the MCO with counterclockwise rings. The diagnosis of the genus was revised by Kritsky et al. (1986), setting species without vaginal sclerite as *incertae sedis*. Since then, additional morphological features have been amended by various authors (Rossim et al., 2011; Rossin & Timi, 2016; Zago et al., 2020). Afterwards, Santos-Neto & Domingues (2023) expanded the understanding of the genus by incorporating molecular data, recognizing as valid several species lacking a vaginal sclerite, previously considered a diagnostic feature, demonstrating that this structure is not a defining characteristic, as *Urocleidoides* species may or may not possess it. Both species of *Urocleidoides* described herein exhibit a copulatory ligament connecting the male copulatory organ to the accessory piece, a feature that has been increasingly reported in recent descriptions (Hasuike et al., 2024) and may be more widespread within the genus than previously recognized.

Although members of Monopisthocotyla are shown to be typically host-specific, *Urocleidoides* exhibits a broader host range, encompassing multiple fish orders. Species of *Urocleidoides* parasitize hosts from three orders and 14 families (Mizelle & Price, 1964; Kritsky et al., 1986; Moreira et al., 2015; Ferreira et al., 2017; Bezerra et al., 2023; Santos-Neto & Domingues, 2023, 2024), indicating a generalist lineage within Dactylogyridae.

Despite this host diversity, morphological constraints remain evident, and some morphologically divergent taxa may still be closely related. Recent studies have emphasized that morphological similarities among species may reflect host-family associations, suggesting convergent adaptations (Bezerra et al., 2023; Santos-Neto & Domingues, 2023; Ebert et al., 2024; Osaki-Pereira et al., 2025). Currently, 62 valid species of *Urocleidoides* are recognized. The genus has been reported in 88 host species across several Neotropical countries, as well as in aquaria in India and the United States (introduced from Neotropical Region).

Overall, this study emphasizes the importance of continued taxonomic investigations of monopisthocotylans in understudied host groups and regions. Such efforts are essential for a more comprehensive understanding of parasite biodiversity, host–parasite associations, and the evolutionary processes shaping these systems. This study provides the first record of Monopisthocotyla in *H. unimaculatus*, with a new genus and species and two new species of *Urocleidoides*.

## Data Availability

No datasets were generated or analysed during the current study.

## Declarations

### AI Statement

ChatGPT was used to review English language.
